# Cortical and Subcortical Circuits for Cross-Modal Plasticity Induced by Loss of Vision

**DOI:** 10.3389/fncir.2021.665009

**Published:** 2021-05-25

**Authors:** Gabrielle Ewall, Samuel Parkins, Amy Lin, Yanis Jaoui, Hey-Kyoung Lee

**Affiliations:** ^1^Solomon H. Snyder Department of Neuroscience, Zanvyl-Krieger Mind/Brain Institute, Johns Hopkins School of Medicine, Baltimore, MD, United States; ^2^Cell, Molecular, Developmental Biology and Biophysics (CMDB) Graduate Program, Johns Hopkins University, Baltimore, MD, United States; ^3^Kavli Neuroscience Discovery Institute, Johns Hopkins University, Baltimore, MD, United States

**Keywords:** cross-modal plasticity, cortical plasticity, cortical circuits, subcortical circuits, sensory loss, multi-sensory interaction, metaplasticity, functional connectivity

## Abstract

Cortical areas are highly interconnected both via cortical and subcortical pathways, and primary sensory cortices are not isolated from this general structure. In primary sensory cortical areas, these pre-existing functional connections serve to provide contextual information for sensory processing and can mediate adaptation when a sensory modality is lost. Cross-modal plasticity in broad terms refers to widespread plasticity across the brain in response to losing a sensory modality, and largely involves two distinct changes: cross-modal recruitment and compensatory plasticity. The former involves recruitment of the deprived sensory area, which includes the deprived primary sensory cortex, for processing the remaining senses. Compensatory plasticity refers to plasticity in the remaining sensory areas, including the spared primary sensory cortices, to enhance the processing of its own sensory inputs. Here, we will summarize potential cellular plasticity mechanisms involved in cross-modal recruitment and compensatory plasticity, and review cortical and subcortical circuits to the primary sensory cortices which can mediate cross-modal plasticity upon loss of vision.

## Introduction

**Figure 1:**
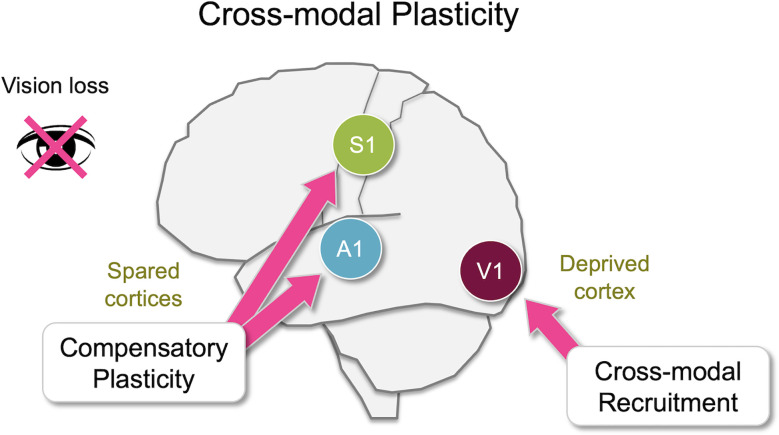
Overview of cross-modal plasticity. Loss of a sensory modality, such as vision, triggers widespread adaptation across different brain areas referred to as cross-modal plasticity. Largely there are two distinct aspects of cross-modal plasticity: cross-modal recruitment and compensatory plasticity. The former involves recruitment of the deprived sensory cortex by the remaining senses, and the latter is manifested as a functional refinement of the spared sensory cortices. While many brain areas are involved in cross-modal plasticity, some of these changes manifest as plasticity at the level of the primary sensory cortices. In this review, we will discuss various cortical and subcortical pathways that are potentially involved in cross-modal plasticity of primary sensory cortices following loss of vision primarily focusing on functional connectivity of the mouse brain.

It is well established that sensory experience can alter cortical and subcortical circuits, especially during early development. In addition, proper sensory experience is crucial for interacting with our environment. Upon loss of a sensory modality, for example, vision, an individual has to rely on the remaining senses to navigate the world. It has been documented that blind individuals show enhanced ability to discriminate auditory (Lessard et al., 1998; Röder et al., 1999; Gougoux et al., 2004; Voss et al., 2004), tactile (Grant et al., 2000; Van Boven et al., 2000) or olfactory (Cuevas et al., 2009; Renier et al., 2013) information. Plastic changes involved can be robust and long–lasting. For example, individuals with congenital bilateral cataracts demonstrate heightened reaction times to auditory stimuli even in adulthood long after surgical removal of cataracts (De Heering et al., 2016). Experimental evidence suggests that there is a rather widespread functional plasticity in the adult sensory cortices upon loss of a sensory modality (Lee and Whitt, 2015), which could constitute the neural basis for cross-modal plasticity (Bavelier and Neville, 2002; Merabet and Pascual-Leone, 2010). Here we use the terminology “cross-modal plasticity” in a broad context to refer to plasticity triggered across sensory modalities to allow adaptation to the loss of sensory input. Changes associated with cross-modal plasticity are often attributed to two distinct plasticity mechanisms that take place across various sensory cortices, some of which manifest at the level of primary sensory cortices (Figure 1). One process is functional adaptation of the primary sensory cortex deprived of its own inputs, which is referred to as “cross-modal recruitment” (Lee and Whitt, 2015) or as “cross-modal plasticity” in its narrower definition (Bavelier and Neville, 2002; Merabet and Pascual-Leone, 2010). The other process, manifested as changes in the functional circuit of the spared sensory cortices, is termed “compensatory plasticity” (Rauschecker, 1995; Lee and Whitt, 2015). A dramatic example of cross-modal recruitment is the activation of visual cortical areas, including the primary visual cortex, when blind individuals are reading braille (Sadato et al., 1996; Buchel et al., 1998; Burton and McLaren, 2006). Compensatory plasticity is observed as functional changes in the circuits of primary auditory and somatosensory cortices of blind individuals (Pascual-Leone and Torres, 1993; Sterr et al., 1998a, b; Elbert et al., 2002). The former is thought to enhance the processing of the remaining senses by recruiting the deprived sensory cortex for increasing the capacity of processing the remaining senses, while the latter is thought to allow refinement of the ability of the spared cortices to process the remaining sensory inputs.

**Figure 2:**
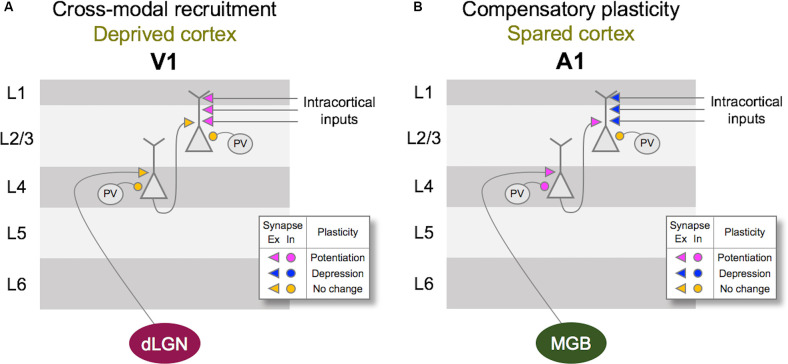
Vision loss triggers cross-modal recruitment and compensatory plasticity across primary sensory cortices. **(A)** Summary of synaptic plasticity observed in V1 following the loss of vision. Synaptic connections that are examined are shown color-coded for potentiation (magenta), depression (dark blue), and no change (yellow) in synaptic strength. Excitatory (Ex) synapses are shown as arrowheads and inhibition (In) synapses are shown as circles. Vision loss does not alter the strength of excitatory feedforward connections from dLGN to Layer 4 (L4) or L4 to L2/3. There is no change in the inhibitory synaptic strength from PV interneurons to L4 or L2/3 principal neurons. In contrast, intracortical synapses onto L2/3 principal neurons potentiate. Based on the fact that L2/3 principal neurons receive multisensory information through long-range intracortical inputs, such adaptation is expected to allow cross-modal recruitment of V1 in the absence of vision. **(B)** Summary of synaptic plasticity observed in the spared A1. Feedforward excitatory synapses from MGBv to L4 as well as L4 to L2/3 potentiate following a week of visual deprivation. This is accompanied by a potentiation of PV inhibition to L4 principal neurons, but not to L2/3 principal neurons. In addition, intracortical excitatory synapses onto L2/3 principal neurons depress. Such synaptic changes are predicted to favor feedforward processing of information at the expense of intracortical influences, and may underlie lowered auditory threshold and refined frequency tuning of A1 L4 neurons following visual deprivation (Petrus et al., 2014).

At the neural level, depriving vision leads to specific adaptation of functional circuits within the primary visual cortex (V1), and a distinct set of changes in the primary auditory (A1) and the primary somatosensory (S1) cortices (Figure 2). As will be discussed in more detail in the subsequent sections, the former involves potentiation of lateral intracortical connections to the principal neurons in the superficial layers of V1 (Petrus et al., 2015; Chokshi et al., 2019), and the latter manifests as potentiation of the feedforward inputs that convey sensory inputs to the cortex (Petrus et al., 2014, 2015; Rodríguez et al., 2018) as well as functional refinement of the cortical circuits (Meng et al., 2015, 2017; Solarana et al., 2019). Cellular mechanisms underlying these two distinct plasticity modes involve both Hebbian and homeostatic metaplasticity as we will describe below, and are thought to be the plasticity of pre-existing functional circuits.

Central to understanding the phenomenon of cross-modal plasticity is the question of what functional circuits allow multisensory information to influence cross-modal recruitment and compensatory changes in primary sensory cortices. The focus of this review will be identifying these potential cortical and subcortical circuits. Most of our discussion will be focused on studies from rodents, which recently have generated cell-type specific data on functional and anatomical connections.

## Cross-Modal Recruitment

Cross-modal recruitment describes the co-opting of a cortical area deprived of its own sensory input by the spared sensory modalities, so that those spared modalities may better guide behavior. While earlier studies have shown such cross-modal recruitment in early-onset blind individuals (Sadato et al., 1996; Buchel et al., 1998; Röder et al., 1999), a more recent study suggests that this can also manifest more acutely in adults. For example, temporarily blindfolding adults while training on braille leads to activation of V1 within a week as visualized in functional magnetic resonance imaging (fMRI; Merabet et al., 2008). Furthermore, this study demonstrated that V1 activity was essential for enhanced learning of braille reading in blindfolded individuals by showing that transcranial magnetic stimulation (TMS) of V1 removes this advantage in blindfolded adults. Cross-modal recruitment is not only restricted to the recruitment of V1 for other senses in blind but has been observed as activation of the auditory cortex by visual stimulation in deaf individuals (Sandmann et al., 2012). Hence such plasticity is thought to be a general principle across sensory cortices. While cross-modal recruitment is viewed as providing adaptive benefits to an individual, it has also been shown to restrict functional recovery of a deprived sense. For example, the success of restoring speech perception in deaf individuals using cochlear implants is inversely correlated with the degree of cross-modal recruitment of A1 by visual inputs (Sandmann et al., 2012).

Cellular and circuit-level plasticity related to cross-modal recruitment can be inferred from studies using various experimental paradigms designed to examine how the deprived cortices change following the loss of their respective sensory modalities. Sensory deprivation paradigms have been traditionally used to examine how sensory experience sculpts the developing sensory cortices. Starting from the initial pioneering work of Hubel and Wiesel, various visual deprivation studies have established the essential role of early visual experience in the proper development of both subcortical and cortical circuits serving visual processing (Hooks and Chen, 2020). While such studies demonstrate that visual cortical plasticity, i.e., ocular dominance plasticity (ODP), is limited to early development termed the “critical period,” the adult visual cortex is not devoid of plasticity. In particular, total deprivation of vision, for example in the form of dark-rearing, has been shown to extend the critical period for ODP (Cynader and Mitchell, 1980; Mower et al., 1981), and the current model is that such deprivation paradigm triggers homeostatic metaplasticity or changes in cortical inhibition to promote Hebbian plasticity involved in ODP (Cooke and Bear, 2014; Hooks and Chen, 2020). Furthermore, total deprivation of vision later in life, in the form of dark-exposure, has been shown to restore ODP in the adult visual cortex (He et al., 2007). At a cellular level, the ability to induce long-term synaptic plasticity, such as long-term potentiation (LTP) and long-term depression (LTD), in sensory cortices is critically dependent on the lamina location of these synapses. For example, across primary sensory cortices, thalamocortical synapses to layer 4 (L4) has an early critical period for plasticity (Crair and Malenka, 1995; Feldman et al., 1998; Jiang et al., 2007; Barkat et al., 2011), but synapses from L4 to L2/3 undergo plasticity through adulthood (Jiang et al., 2007). Interestingly, L2/3 is considered a location where top-down contextual information is provided for sensory processing and has been shown to exhibit modulation of activity by other sensory modalities (Lakatos et al., 2007; Iurilli et al., 2012; Ibrahim et al., 2016; Chou et al., 2020). L2/3 is a logical substrate for cross-modal recruitment because of its susceptibility to adult plasticity and its role in integrating top-down multisensory inputs.

### Plasticity of V1 Circuit That Can Support Cross-modal Recruitment

Vision loss alters the strength of both excitatory and inhibitory synaptic transmission on V1 L2/3 principal neurons. Experiments in rodents have demonstrated that even as little as 2 days of visual deprivation leads to the strengthening of excitatory synapses observed as increases in the average amplitude of miniature excitatory postsynaptic currents (mEPSCs; Desai et al., 2002; Goel and Lee, 2007; Maffei and Turrigiano, 2008; Gao et al., 2010; He et al., 2012; Chokshi et al., 2019). This plasticity, which was initially interpreted as a form of *in vivo* synaptic scaling (Desai et al., 2002; Goel and Lee, 2007), is observed around the 3rd postnatal week (Desai et al., 2002; Goel and Lee, 2007) and persists through adulthood (Goel and Lee, 2007; Petrus et al., 2015). However, strengthening of excitatory synapses by visual deprivation is dependent on the mode of visual deprivation, such that total loss of vision is necessary, and it is not observed with bilateral lid-suture (He et al., 2012). Lid-suture is different from other modes of visual deprivation, such as dark-exposure, enucleation, or intraocular tetrodotoxin (TTX) injection, in that visual stimuli through the closed eyelids can elicit visually evoked potentials (VEPs) in V1 (Blais et al., 2008). This suggests that residual vision through the closed eyelids is sufficient to prevent visual deprivation-induced synaptic scaling. Sensory deprivation-induced strengthening of excitatory synapses is not restricted to V1 L2/3 but is observed in A1 L2/3 following a conductive hearing loss (Kotak et al., 2005). Interestingly, whisker deprivation is typically unable to increase the strength of excitatory synapses in barrel cortex L2/3 neurons (Bender et al., 2006; He et al., 2012; Li et al., 2014; see Glazewski et al., 2017 for exception) which suggests that whisker deprivation may be similar to lid-suture in that it may not completely remove all inputs to the barrel cortex.

In addition to the plasticity of the excitatory synapses, inhibitory synapses on principal neurons in V1 also undergo lamina-specific adaptation to visual deprivation, which differs depending on the developmental age. In V1 L4 of rodents, monocular deprivation before the critical period leads to a reduction of inhibition, measured as a decrease in both spontaneous and evoked inhibitory postsynaptic currents (IPSCs) in the deprived monocular zone of V1 (Maffei et al., 2004), whereas monocular deprivation during the critical period leads to an increase in inhibition (Maffei et al., 2006; Nahmani and Turrigiano, 2014). With L4 serving as the main thalamorecipient layer, this increase in inhibition within L4 later in development could serve to lower the recurrent activity and reduce the propagation of sensory information in V1. In L2/3, a few days of visual deprivation during the critical period leads to a reduction in the frequency of miniature inhibitory postsynaptic currents (mIPSCs; Gao et al., 2010, 2014). This decrease in mIPSC frequency correlated with a reduction in the density of perisomatic GAD65 punta (Gao et al., 2014) suggesting a decrease in the number of inhibitory synaptic contacts likely from local parvalbumin-positive (PV) interneurons. However, visual deprivation-induced plasticity of inhibitory synapses in the adult V1 L2/3 is different in that it is specific to action potential-independent inhibitory synaptic transmission (Barnes et al., 2015; Gao et al., 2017), which suggests that it is not likely due to changes in the number of inhibitory synapses. The selective plasticity of action potential independent mIPSCs is thought to benefit sensory processing in the mature cortex by maintaining temporal coding while providing homeostasis of overall neural activity (Gao et al., 2017).

**Figure 3:**
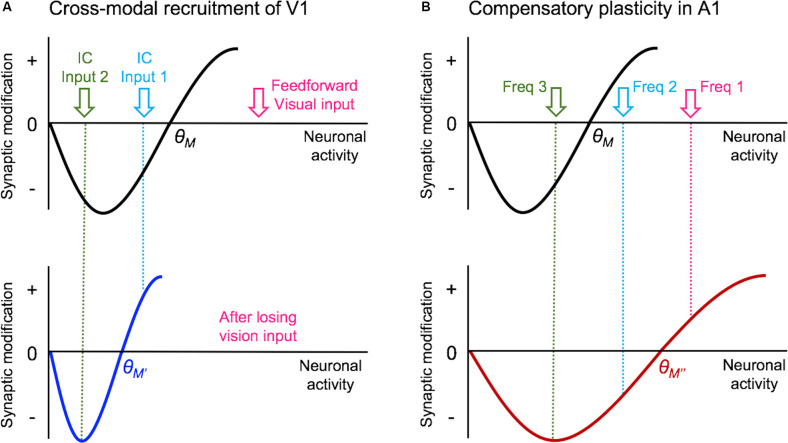
Metaplasticity model for cross-modal synaptic plasticity induced by vision loss. The sliding threshold (or BCM) model of metaplasticity posits that the synaptic modification threshold (θ_M_) for Long-term potentiation (LTP) and Long-term depression (LTD) slides as a function of past activity (Cooper and Bear, 2012). **(A)** In V1, loss of vision is expected to reduce the θ_M_ to a new value (θ_M’_), which will favor LTP induction. This will allow some of the stronger intracortical inputs (IC Input 1) to cross the threshold and potentiate. However, the weaker intracortical inputs (IC Input 2) will still fall below the θ_M’_ value and remain weaker. Such plasticity is expected to allow V1 neurons to preferentially respond to IC Input 1 in the absence of vision. As many of these intracortical inputs are multisensory, such as feedback projections from HVAs and other cortico-cortical connections, selective potentiation of intracortical synapses could allow V1 to process non-visual contextual information. **(B)** In the spared primary sensory cortex, as given an example of A1, loss of vision is thought to increase the synaptic modification threshold (θ_M”_) based on the observation that there is potentiation of feedforward excitatory inputs originating from MGBv. The resulting metaplasticity is expected to sharpen the response properties of A1 neurons, such that the strength of inputs carrying two close sound frequencies (Freq 1 and Freq 2) will separate further by a preferential strengthening of the most dominant frequency (Freq 1).

In terms of the mode of plasticity, initial studies have interpreted the overall increase in mEPSC amplitudes following visual deprivation in the framework of synaptic scaling (Desai et al., 2002; Goel and Lee, 2007). However, recent data suggest that the changes are not global across all synapses but are input-specific and restricted mainly to intracortical synapses without changes in the feedforward input from L4 (Petrus et al., 2015; Figure 2A). Furthermore, the increase in mEPSC amplitudes with visual deprivation requires NMDA receptor (NMDAR) activation (Rodríguez et al., 2018), which distinguishes it from synaptic scaling which has been shown not to require the activity of NMDARs (O’Brien et al., 1998; Turrigiano et al., 1998). On the contrary, experimental evidence suggests that synaptic scaling induced by inactivity is accelerated when blocking NMDARs (Sutton et al., 2006). The observation that visual deprivation-induced potentiation of excitatory synapses in V1 L2/3 is input-specific and dependent on NMDAR activity suggests that it is likely a manifestation of Hebbian LTP following metaplasticity as proposed by the Bienenstock-Cooper-Monroe (BCM) model (Bienenstock et al., 1982; Bear et al., 1987; Cooper and Bear, 2012; Lee and Kirkwood, 2019; Figure 3). The BCM model, often referred to as the “sliding threshold” model, posits that the synaptic modification threshold for LTP/LTD induction “slides” is a function of the past history of neural activity. An overall reduction in neural activity, as would occur in V1 following visual deprivation, is expected to lower the synaptic modification threshold to promote LTP induction. Indeed, studies have demonstrated that visual deprivation can lower the LTP induction threshold in V1 L2/3 (Kirkwood et al., 1996; Guo et al., 2012). However, to induce LTP with the lowered synaptic modification threshold, synaptic activity is required. While visual deprivation reduces the overall activity in V1, a recent study reported that spontaneous activity is increased following a few days of visual deprivation in the form of dark exposure (Bridi et al., 2018). In addition, the study demonstrated that this increase in spontaneous activity is critical for strengthening excitatory synapses on V1 L2/3 neurons dependent on the activity of the GluN2B subunit of NMDARs (Bridi et al., 2018). It is possible that visual deprivation-induced reduction in the inhibitory synaptic transmission (Gao et al., 2010, 2014; Barnes et al., 2015) may contribute to enhance spontaneous activity or help facilitate the induction of LTP. Collectively, these studies suggest a novel model in which visual deprivation reduces the threshold for LTP induction, and the increase in spontaneous activity acts on NMDARs to trigger potentiation of excitatory synapses, which tend to be of intracortical origin. Therefore, understanding the potential source of these intracortical synapses to V1 L2/3 will provide insights into how V1 may undergo cross-modal recruitment in the absence of vision.

**Figure 4:**
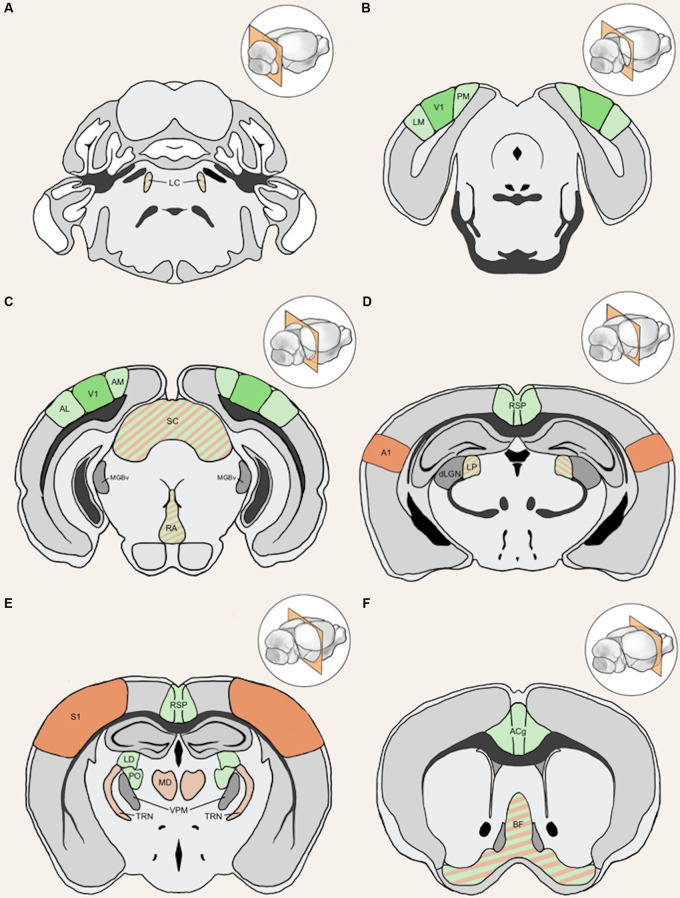
Anatomical structures implicated in cross-modal plasticity induced by vision loss. Six coronal sections of a mouse brain are listed in order from posterior to anterior. Structures involved in cross-modal recruitment are labeled in green (V1, LM, PM, AM, AL, RSP, ACg, LD, PO), structures involved in compensatory plasticity are labeled in orange (A1, S1, MD, TRN), and those involved in both are labeled with stripes of green and orange (LC, superior colliculus (SC), RA, LP, BF). Darker shades (V1, A1, S1) represent cortical structures that have been experimentally demonstrated to undergo plasticity with visual deprivation, while lighter shades are tentative structures implicated in the plasticity. Primary sensory thalamic nuclei are labeled in gray (dLGN, MGBv, VPM). Inset in each panel shows the location of the coronal section plane. **(A)** The locus coeruleus (LC) contains the cell bodies of most norepinephrine expressing neurons. These cells send vast projections across cortical areas and are involved in both attention and arousal. Following vision loss, the increased salience of auditory and somatosensory cues might be conveyed through norepinephrine projections, facilitating potentiation in spared sensory cortices (compensatory plasticity) as well as potentiation of spared inputs into V1 (cross-modal recruitment). The relative concentration of norepinephrine is thought to play a role in determining the polarity spike-timing-dependent of plasticity (STDP; Seol et al., 2007). **(B)** The lateral medial visual area (LM) and the posteromedial visual area (PM) are both HVAs, which flank V1. HVAs process higher-order visual information and provide feedback connections to V1 which modulate V1 activity. Visual deprivation leads to plasticity specifically of intracortical inputs in L2/3 pyramidal neurons without changes in the strength of feedforward inputs from the thalamus to L4 or from L4 to L2/3 (Petrus et al., 2014, 2015; Chokshi et al., 2019; see Figure 2A). **(C)** This section shows V1 in addition to the anterolateral visual area (AL) and the anteromedial visual area (AM), which are both a part of the HVA. The section also includes the SC, the primary auditory thalamus (MGBv), and the raphe nuclei (RA). SC is an area of the brain that is in charge of processing sensory input and is involved in the integration of visual, auditory, and tactile stimuli, hence could play a role in cross-modal plasticity. MGBv transmits auditory information to A1. Visual deprivation induces potentiation of MGBv synapses to A1 L4 principal neurons (Petrus et al., 2014; see Figure 2B). RA is found in the brain stem and contains serotonergic neurons. Serotonin is implicated in cross-modal recruitment of V1 (Lombaert et al., 2018) and compensatory plasticity of S1 (Jitsuki et al., 2011) following visual deprivation. **(D)** This section contains the lateral posterior thalamic nucleus (LP), the retrosplenial cortex (RSP), the primary visual thalamus (dLGN), and the primary auditory cortex (A1). LP is a higher-order visual thalamus in rodents, which is equivalent to the pulvinar in primates. LP receives input from SC and influences V1, and it has been shown to reduce background noise to enhance visual responses (Fang et al., 2020). SC to LP circuit mainly targets inhibitory neurons in L1 of V1 (Fang et al., 2020). RSP is interconnected with the lateral dorsal nucleus of thalamus (LD; Shibata, 2000). LD is a higher-order thalamic nucleus that plays a part in learning and memory and may transmit somatosensory information to V1. A1 processes auditory information and undergoes compensatory plasticity in the absence of vision (Goel et al., 2006; Petrus et al., 2014, 2015; Meng et al., 2015, 2017; Solarana et al., 2019; see Figure 2B). **(E)** The retrosplenial cortex (RSP) along with the mediodorsal nucleus of the thalamus (MD), the thalamic reticular nucleus (TRN), and the primary somatosensory cortex (S1) are highlighted. RSP is a multisensory cortical area that sends projections to V1 (see Figure 5). MD is a higher-order thalamic nucleus that is reciprocally connected with the prefrontal cortex and projects to TRN. MD is involved in attention and learning by gating sensory inputs. TRN is a band of inhibitory neurons that provides the major corticothalamic feedback inhibition to the primary sensory thalamic nuclei. Hence, TRN is in an ideal position to regulate feedforward excitatory thalamocortical input to A1 and S1 to mediate compensatory plasticity. S1 processes tactile information and undergoes compensatory plasticity in the absence of vision (Goel et al., 2006; Jitsuki et al., 2011; He et al., 2012). **(F)** The basal forebrain (BF) and the anterior cingulate cortex (ACg) are highlighted in this section. BF includes structures involved in the production of acetylcholine, including the nucleus basalis and medial septum, which affects attention and plasticity. ACg is a multisensory cortex that has direct and indirect functional connections to V1 (see Figure 5).

In the following sections, we will review potential cortical and subcortical structures that can mediate cross-modal plasticity observed with vision loss. The anatomical locations of these structures are highlighted in Figure 4. First, we will provide information on potential functional circuits involved in cross-modal recruitment of V1, which involve cortico-cortical connections from multisensory or spared sensory cortices. Some of these cortical interactions involve indirect functional circuits mediated by subcortical structures. In addition, we will outline various neuromodulatory systems, which can enhance or enable plasticity of these intracortical and subcortical inputs to V1.

### Cortical Inputs to V1 L2/3 That Can Mediate Cross-modal Recruitment

**Figure 5:**
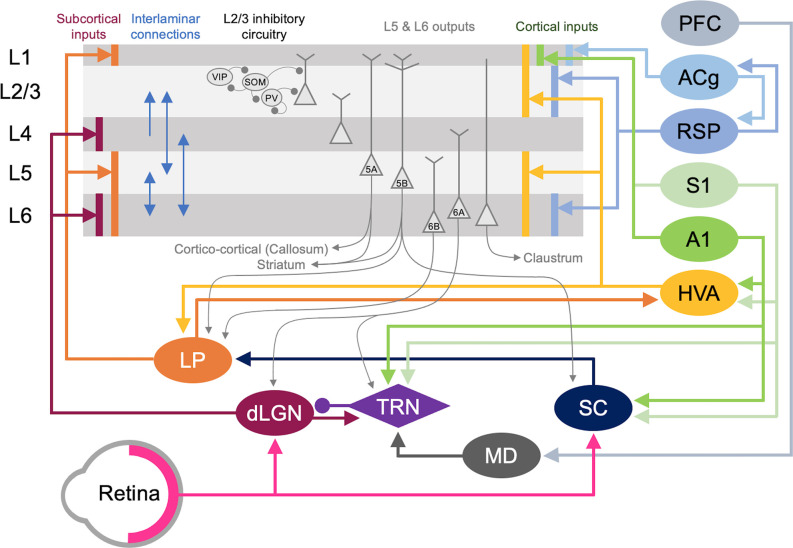
Cortical and subcortical circuits for multisensory influence on V1. The laminar profile of subcortical inputs from dLGN and LP to V1 is shown on the left. Major interlaminar excitatory connections are shown next in blue arrows followed by the inhibitory local circuit in L2/3. Next, the major outputs of L5 and L6 neurons are shown. At the rightmost side, the origins and laminar profiles of cortical inputs to V1 are shown. Subcortical structures are shown below V1 and cortical structures are listed on the right side. Arrows (→) depict excitatory inputs and inputs ending in a round circle (—•) show inhibitory connections. The extent of the spread of inputs to V1 that span different laminae are depicted as vertical bars. V1 L2/3 and L5A neurons form reciprocal connections with HVA neurons (Kim et al., 2015; Glickfeld and Olsen, 2017), which is omitted in the figure for clarity. Direct cortico-cortical connections that can provide multisensory information to V1 originate from HVA, A1, S1, RSP, and ACg. In addition, as depicted in the figure many of the subcortical and cortical structures form cortico-thalamo-cortical loops that can provide multisensory influence on V1: for example, HVA–LP–V1, PFC–MD–TRN–dLGN–V1, S1/A1–TRN–dLGN–V1, and S1/A1–SC–LP–V1.

V1 L2/3 cells are a probable substrate for multimodal recruitment of V1 due to their extensive and varied inputs. Intracortical inputs onto L2/3 of V1 originate from various sources, including local connections from within V1, feedback projections from higher-order visual areas (HVAs), other sensory cortices, as well as other cortical areas (e.g., Wertz et al., 2015; Figure 5). A recent monosynaptic tracing of presynaptic partners of a single V1 L2/3 pyramidal neuron showed that these neurons receive inputs from 70 to 800 neurons across many brain regions with the majority of them (50–700 neurons) situated within V1 (Wertz et al., 2015). In addition to these local inputs, V1 L2/3 neurons receive multisensory information from other cortical areas via direct long-range intracortical connections, as well as indirectly via subcortical structures (Figure 5; “Subcortical Sources of Inputs to V1 L2/3 That Can Mediate Cross-modal Recruitment” section). Therefore, V1 L2/3 could mediate a role in cross-modal recruitment in the absence of vision.

Cortical inputs that reside locally within V1 serve as the major source of excitatory inputs onto L2/3 neurons with local L2/3 inputs being the most numerous (Binzegger et al., 2004; Wertz et al., 2015) with connections heavily favored between neurons showing similar functional properties (Ko et al., 2011; Wertz et al., 2015; Lee et al., 2016). Neurons across the various layers are interconnected to allow for the efficient processing of information. Visual information is transmitted from the primary visual thalamus (dLGN), which densely projects onto V1 L4 neurons. L4 principal neurons relay this information across V1, but most prominently onto L2/3 neurons (Binzegger et al., 2004; Wertz et al., 2015). L5 is mainly an output layer, projecting to HVAs, the contralateral cortex, the striatum, the higher-order thalamus, and other subcortical targets, but it also projects locally within V1 to L2/3 (Binzegger et al., 2004; Kim et al., 2015; Ramaswamy and Markram, 2015; Wertz et al., 2015). L5 neurons integrate inputs from a variety of sources, including local inputs from L4 and L2/3 (Binzegger et al., 2004; Wertz et al., 2015) as well as feedback projections from HVAs and multisensory cortical areas such as the retrosplenial cortex (Kim et al., 2015). The output from lower L5 (L5b) to higher-order visual thalamus (lateral posterior nucleus, LP; Kim et al., 2015; Roth et al., 2016) allows indirect communication from V1 to HVA forming a transthalamic or cortico-thalamo-cortical loop (Sherman, 2016). L6 is a thalamorecipient layer, like L4, and also receives local inputs from L2/3, L4, and L5 as well as feedback projections from HVAs (Thomson, 2010). A subset of L6 neurons, which are identified by the marker NTSR1 (Gong et al., 2007), project back to the dLGN to provide corticothalamic feedback (Olsen et al., 2012; Bortone et al., 2014; Sundberg et al., 2018), which also involves disynaptic inhibition through the thalamic reticular nucleus (TRN; Olsen et al., 2012). Corticothalamic L6 neurons also project locally within V1, and it has been observed that they may provide net inhibition to the other layers (Olsen et al., 2012; Bortone et al., 2014) *via* recruitment of L6 fast-spiking interneurons with translaminar projections (Bortone et al., 2014). While local connectivity within V1 serves to process visual information, it can also convey multisensory information to L2/3. In particular, infragranular layers receive multisensory information from other cortical and subcortical areas (Thomson, 2010; Kim et al., 2015).

A second major source of cortical inputs to V1 L2/3 is feedback connections from HVAs (Wertz et al., 2015). In higher mammals, including humans and primates, HVAs integrate and process higher-order visual information, such as form and movement of objects (Orban, 2008). In rodents, 10 HVAs are anatomically identified, using intrinsic signal imaging, surrounding V1 (Garrett et al., 2014; Glickfeld and Olsen, 2017). While in primates and carnivores, HVAs are mostly hierarchically organized such that the main feedback to V1 is from the secondary visual cortex (V2, area 18; Felleman and Van Essen, 1991), in rodents each HVA is highly interconnected with V1 and send direct feedback projections to V1 (Glickfeld and Olsen, 2017). Direct cortico-cortical feedback connections from HVAs originate in L2/3 and L5 and arrive through L1, L2/3 as well as L5/6 of V1 (Glickfeld and Olsen, 2017). These feedback connections from HVAs have been shown to synapse onto pyramidal neurons as well as PV interneurons (Johnson and Burkhalter, 1996; Yang et al., 2013; Lu et al., 2014), thereby recruiting both the excitatory and inhibitory networks in V1 with a functional bias towards excitation (Shao and Burkhalter, 1996). In rodents, HVA neurons that provide feedback to V1 are reciprocally connected to HVA projecting V1 neurons in L2/3 (Johnson and Burkhalter, 1997), forming a closed-loop circuit which may amplify the feedback control of V1 (Glickfeld and Olsen, 2017). In addition to direct cortico-cortical connections, HVAs and V1 are indirectly connected via the higher-order thalamus. For example, HVAs send feedforward projections to the pulvinar (lateral posterior nucleus, LP, in rodents), a higher-order visual thalamus, which then sends projections to L1 and deeper layers of V1 (Roth et al., 2016; Zhou et al., 2017; Fang et al., 2020). Hence, HVAs can influence V1 processing via both cortico-cortical and indirect cortico-thalamo-cortical feedback loops.

The influence of HVA feedback connections in V1 is highlighted by a phenomenon called the perceptual “filling-in” effect (Weil and Rees, 2011). Individuals with a focal scotoma will perceive the missing visual space as being “filled-in” such that the person is often unaware of the scotoma (Bender and Teuber, 1946). Because this “filled-in” percept contains higher-order visual features, such as texture, the information is thought to originate from HVAs (Ramachandran and Gregory, 1991; Zur and Ullman, 2003). Recent studies using rodents also have shown that V1 neurons can respond to higher-order visual features in awake preparations and that these responses are dependent on feedback connections from HVAs as demonstrated using optogenetic silencing (Keller et al., 2020; Pak et al., 2020). In addition to feedforward information originating from V1, HVAs receive multisensory information *via* connections from other sensory cortices (Gamanut et al., 2018). For example, V2L, which is an HVA lateral to V1 corresponding to anterolateral area (AL; Meijer et al., 2020) and lateromedial (LM; Sanderson et al., 1991), receive connections from both V1 and A1 (Laramee et al., 2011). A1 projections to V2L mainly terminate in supra- and infragranular layers (Laramee et al., 2011). L5 neurons in V2L provide major feedback to V1 (Bai et al., 2004) and receive direct inputs from A1 on their apical and basal dendrites (Laramee et al., 2011), thus demonstrating an A1-V2L-V1 pathway. The rostrolateral area (RL), another HVA in rodents, has been shown to receive tactile information from S1 as verified through whole-cell recordings and tracing studies (Olcese et al., 2013). Therefore, feedback projections from HVAs can relay other sensory information to V1.

In addition to the indirect route through HVAs, other sensory modalities can also gain access to V1 via direct connections (Figure 5). Anatomical tracing studies have demonstrated direct cortico-cortical projections from A1 (Iurilli et al., 2012; Wertz et al., 2015; Ibrahim et al., 2016; Deneux et al., 2019) and S1 (Wertz et al., 2015), especially to the superficial layers of V1. Recent studies showed that these projections are functional and can influence V1 processing (Iurilli et al., 2012; Ibrahim et al., 2016; Deneux et al., 2019). Ibrahim and colleagues (2016) found that sound increases the spike rate and sharpens orientation selectivity of V1 L2/3 neurons. This study further demonstrated that sound activates a disinhibitory circuit in L1 and L2/3 involving vasoactive intestinal peptide-positive (VIP) and somatostatin-positive (SOM) interneurons, which is mediated by a direct functional connection from A1 L5 that arrives through V1 L1 (Ibrahim et al., 2016). A1 neurons also have been shown to project directly to PV interneurons in V1 (Lu et al., 2014; Ibrahim et al., 2016), however, PV neuronal responses are not effectively altered by sound (Ibrahim et al., 2016). Interestingly, the influence of A1 on V1 appears to be context-dependent. A1 projections to V1 have a net excitatory effect in the presence of visual stimuli but a net inhibitory effect in the absence of visual stimuli (Deneux et al., 2019). These projections predominantly originate from A1 L5 neurons encoding loud sound (Deneux et al., 2019). The role of SOM inhibitory circuit in cross-modal recruitment is also evident with monocular enucleation paradigm (Scheyltjens et al., 2018), where the deprived monocular zone of V1 becomes reactivated by whisker inputs (Van Brussel et al., 2011). In addition to the inhibitory circuit within L2/3 of V1, L1 inhibitory neurons can also provide multisensory influence on V1 functionality. For example, L1 inhibitory neurons contain a subpopulation of neurons that respond to whisker touch (Mesik et al., 2019). Multisensory influence on neural activity is not limited to V1: whisker stimulation and visual stimulation produce subthreshold responses in A1, and likewise, auditory stimulation and visual stimulation produce subthreshold responses in S1 (Iurilli et al., 2012; Maruyama and Komai, 2018). Subthreshold influence on primary sensory cortical activity by other sensory modalities is not just restricted to rodents but has also been reported in awake primates (Lakatos et al., 2007). While there are direct anatomical pathways between primary sensory cortices in primates (Falchier et al., 2002; Cappe and Barone, 2005), the somatosensory evoked oscillations in L2/3 of A1 are thought to occur via subcortical inputs based on their short latency (Lakatos et al., 2007). Such subcortical sources will be discussed in the next section. Overall, cross-modal influence seems to be a general property of primary sensory cortices across species.

Multisensory cortical regions serve as another source through which V1 can be recruited by other sensory modalities after the loss of vision (Figure 5). One such region is the anterior cingulate cortex (ACg). Using tracing methods, it was shown that ACg neurons contain two distinct populations, L2/3, and L5 neurons that project directly to V1 and neurons primarily in L5 that project to the superior colliculus (SC; Zhang et al., 2016). Consistent with this anatomy, ACg has been shown to directly (Zhang et al., 2016) and indirectly (Hu et al., 2019) modulate the activity of V1 neurons. Optogenetic activation of ACg axons elicits a short latency monosynaptic EPSC and a longer latency disynaptic IPSC in V1 L2/3 neurons (Zhang et al., 2014), which illustrates recruitment of both excitatory and inhibitory networks. There are two indirect routes through which the ACg exerts its modulatory activity on V1 neurons. The first is through the SC and the posterior lateral posterior nucleus of the thalamus (pLP; ACg-SC-pLP-V1) and the second via the anterior LP (ACg-aLP-V1; Hu et al., 2019). Activating both pathways enhances visual behavior as well as responses in V1 neurons (Hu et al., 2019). While LP receives inputs from ACg and projects to V1, whether the ACg recipient LP neurons are the ones projecting to V1 is unclear. A recent study suggests that ACg projects to medial LP (mLP), which does not project directly to V1, but to HVAs (AL, RL, AM, PM; Bennett et al., 2019). Since the HVAs project to V1, this suggests a more indirect pathway in which ACg could influence V1 function.

The retrosplenial cortex (RSP) is another multisensory area directly linked to V1. Neurons from the RSP were shown to directly synapse unto V1 L2/3 neurons (Wertz et al., 2015) and L6 cortico-thalamic neurons (Vélez-Fort et al., 2014). These V1 projecting RSP neurons were also shown to be responsive to rotation implicating them as a potential source of head-related motion signals to V1 (Vélez-Fort et al., 2014). The RSP also received inputs directly from A1 and indirectly from S1 through the claustrum (Todd et al., 2019). RSP also forms reciprocal cortico-cortical connections between ACg and V1 (ACg–RSP–V1; Zhang et al., 2016). The influence of multisensory cortex on sensory processing is not limited to V1. Pairing of a tone with the activation of the frontal cortex leads to enhanced frequency selectivity and functional organization in A1 neurons (Winkowski et al., 2018).

Recently, posterior parietal cortex (PPC) has been suggested to play a role in cross-modal recruitment (Gilissen and Arckens, 2021). This is based on the multisensory nature of PPC and its functional modulation of V1 (Hishida et al., 2018). Recent studies demonstrated that PPC is involved in resolving sensory conflict during auditory-visual discrimination tasks (Song et al., 2017) and is involved in transferring sensory-specific signals to higher order association areas (Gallero-Salas et al., 2021). RL and AM, two HVAs, are considered part of the PPC because they display connectivity patterns similar to other components of the PPC (Gilissen et al., 2021).

### Subcortical Sources of Inputs to V1 L2/3 That Can Mediate Cross-modal Recruitment

In addition to inputs from cortical areas, V1 also receives multimodal information from various subcortical regions (Figure 5). The lateral posterior nucleus (LP), posterior thalamic nucleus (PO), and lateral dorsal nucleus of the thalamus (LD) all project directly to V1 and might be potential sources of multimodal input subserving cross-modal recruitment.

The higher-order visual thalamus, called the lateral posterior nucleus (LP) in rodents, is equivalent to the pulvinar in primates (Baldwin et al., 2017; Zhou et al., 2017). A recent study suggests that LP can be subdivided into three portions based on connectivity: (1) posterior-dorsal LP (pLP) receives input primarily from SC and HVAs which are considered the “ventral stream” equivalent in rodents (LI, POR); (2) anterior-ventral LP (aLP) receives input primarily from V1 and HVAs considered the “dorsal stream” (AL, RL, AM, PM); and (3) mLP with inputs from frontal cortical areas (ACg and orbitofrontal; Bennett et al., 2019). Most of the projections to LP are reciprocal, but they also form a cortico-thalamo-cortical loop (Sherman, 2016) to connect different cortical areas. Cortical inputs to LP originate from L5/6 of the cortical areas (Roth et al., 2016). The major subcortical input to LP is from the SC (Ibrahim et al., 2016; Roth et al., 2016; Zingg et al., 2017), which integrates multisensory information and is implicated in spatial attention (Krauzlis et al., 2013). Superficial layers of SC receive visual information from both V1 and retina (Krauzlis et al., 2013; Zingg et al., 2017; Cang et al., 2018), while intermediate and deep layers receive multimodal inputs (Krauzlis et al., 2013; Cang et al., 2018) and inputs from HVAs (Krauzlis et al., 2013). LP projects to L4 of HVAs and predominantly to L1 and deep layers of V1 (Roth et al., 2016; Zhou et al., 2017; Bennett et al., 2019). Hence, LP is in a position to influence V1 processing either directly or indirectly through HVAs. It was recently demonstrated in rodents that LP provides contextual information to V1, especially pertaining to distinguishing self-generated motion, and information from a wider visual field from that of local V1 neurons (Roth et al., 2016). In addition, it was reported that LP acts to enhance V1 L2/3 responses by subtracting “noisy” background information from visual stimuli (Fang et al., 2020). This effect was shown to occur *via* a bottom-up alternative pathway originating from the retina that routes through SC to LP, which then makes functional connections to inhibitory neurons in V1 L1 (Fang et al., 2020). Based on the multisensory information it receives *via* SC, it is possible that LP inputs may provide other sensory information to V1 in the absence of vision. In support of this idea, a recent study demonstrated that LP conveys visual information arising from SC to A1 (Chou et al., 2020). In particular, it was shown that this subcortical circuit allows a visual looming stimulus, which produces an innate fear response in mice (Yilmaz and Meister, 2013), to sharpen frequency tuning and increase the signal to noise ratio of auditory responses in L2/3 of A1 (Chou et al., 2020). It was demonstrated that SC-LP input to A1 activates inhibitory neurons in L1 as well as PV interneurons in L2/3 (Chou et al., 2020). It is interesting to contrast this with the previously discussed enhancement of tuning and signal-to-noise ratio in V1 L2/3 with sound, which involved direct input from A1 L5 (Ibrahim et al., 2016). Whether similar indirect influence through LP can provide cross-modal modulation of V1 responses remains to be determined.

The posterior thalamic nucleus (PO) and lateral dorsal nucleus of thalamus (LD) also project directly to V1 (van Groen and Wyss, 1992; Charbonneau et al., 2012). PO is a higher-order somatosensory relay nucleus, hence its direct projection to V1 could become a channel for providing somatosensory information and form the basis for cross-modal recruitment following vision loss. In addition, PO has direct projections to several HVAs (Sanderson et al., 1991; Olcese et al., 2013), which might mediate indirect influence on V1. LD is extensively interconnected with RSP (Shibata, 2000) and the hippocampal formation (Todd et al., 2019), and LD contains head direction cells that require visual inputs (Mizumori and Williams, 1993). These findings have led to the characterization of LD as a higher-order thalamic nucleus involved in learning and memory. More recently, the finding that neurons in LD respond to whisker stimulation (Bezdudnaya and Keller, 2008) suggests that LD might relay somatosensory information to V1.

### Neuromodulatory Influences on Cross-modal Recruitment

As described above, there are numerous sources of cortical and subcortical input to V1 that could serve as substrates for allowing other sensory systems to recruit V1. One key plasticity mechanism that can aid in the cross-modal recruitment is the potentiation of the lateral intracortical inputs to V1 L2/3 observed following several days of total visual deprivation (Petrus et al., 2015). This particular study did not identify the source of these glutamatergic intracortical inputs, and these synapses were defined as intracortical based on exclusion criteria that they were not from L4 (Petrus et al., 2015). Hence, in addition to “true” intracortical inputs carrying multisensory information, they could also include subcortical excitatory synapses described above. The functional consequence of potentiating these intracortical excitatory synapses is that it would allow the normally subthreshold multisensory influences to potentially cross the action potential threshold to recruit the dormant V1 for processing information from the intact senses. As discussed in a previous section (“Plasticity of V1 Circuit That Can Support Cross-modal Recruitment” section), the synaptic plasticity mechanism that is thought to allow potentiation of these intracortical synapses is likely a reduction in the synaptic modification threshold via metaplasticity triggered by the loss of visually evoked activity in V1. As intracortical inputs would retain activity driven from the intact senses, it is possible that their activity would cross the lowered synaptic modification threshold to produce NMDAR-dependent LTP (Figure 3A). However, in addition to the lowered synaptic modification threshold, other factors might be at play to enhance the plasticity of the intracortical inputs.

Neuromodulators such as acetylcholine, norepinephrine, and serotonin play a key role in facilitating plasticity (Gu, 2002). In V1 L2/3, norepinephrine and acetylcholine are involved in sharpening spike timing-dependent plasticity (STDP), and their relative concentrations are thought to determine the polarity of STDP (Seol et al., 2007; Huang et al., 2012). While the initial studies showed that activation of beta-adrenergic receptors and muscarinic acetylcholine receptors (mAchRs) are respectively critical for LTP and LTD, it is now clear that this effect is due to the differential coupling of these receptors to downstream second messenger signaling. Regardless of the neuromodulators, activation of cAMP-coupled receptors is critical for LTP while phospholipase C (PLC)-coupled receptors are involved in LTD (Huang et al., 2012). Both norepinephrine and acetylcholine have been shown critical for *in vivo* sensory experience-dependent plasticity, as they are necessary for (Bear and Singer, 1986; Imamura and Kasamatsu, 1989) and can accelerate (Hong et al., 2020), ocular dominance plasticity in V1. Norepinephrine and acetylcholine are associated with arousal and attention, hence if they are involved in cross-modal plasticity, it would suggest that behavioral state would be a variable in engaging the cellular mechanisms of plasticity.

Serotonin has received some attention as promoting plasticity in the adult brain. The role of serotonin in sensory perception has been historically revealed through studies of hallucinogenic serotonin receptor agonists such as LSD and psilocybin, but recent studies highlight its role in adult cortical plasticity. For example, administration of a serotonin reuptake inhibitor, fluoxetine, was found to reinstate ODP in adult V1 of rats (Maya Vetencourt et al., 2008). This suggests that juvenile forms of plasticity could be enabled in the adult brain by serotonin. Of interest, serotonin has also been specifically implicated in cross-modal recruitment in adults. Lombaert et al. (2018) found evidence that serotonin tone is higher in the deprived V1 using a monocular enucleation paradigm, and that serotonin facilitates recruitment of the deprived V1 by whisker stimulation (Lombaert et al., 2018). In particular, long–term cross-modal recruitment was dependent on activation of 5HT-2A and 5HT-3A receptors as determined by specific antagonists.

At the circuit level, neuromodulators, in particular serotonin and acetylcholine, act through VIP interneurons in the superficial layers of V1 (Tremblay et al., 2016), which is the same circuit element that allows cross-modal modulated of V1 by sound (Ibrahim et al., 2016). Coincidently, VIP interneurons are a subset of 5HT-3A receptor expressing inhibitory interneurons (Tremblay et al., 2016), which may explain the dependence of cross-modal recruitment on 5HT-3A receptors (Lombaert et al., 2018). Collectively, these findings suggest that VIP interneuron-mediated disinhibitory circuit may be a common element for gating cross-modal information flow into L2/3 of V1 to mediate cross-modal recruitment.

## Compensatory Plasticity

In addition to cross-modal recruitment of V1, which may add capacity to the processing of the remaining senses, there is evidence that the cortical areas serving the spared senses also undergo their own unique adaptation to enhance the processing of their sensory inputs. This phenomenon is referred to as “compensatory plasticity” (Rauschecker, 1995; Lee and Whitt, 2015; Figure 1). Such compensatory changes are seen in parts of the cortex serving both somatosensation and audition. Blind individuals who use a single finger to read Braille exhibit increased representation of that reading finger in the sensorimotor cortex compared to nonreading fingers and compared to sighted controls (Pascual-Leone and Torres, 1993). The auditory cortex likewise undergoes expansion as measured by magnetic source imaging (Elbert et al., 2002). In early blind subjects, the response levels of auditory cortical neurons differ from sighted controls, and these changes are interpreted as supporting more efficient processing of auditory information (Stevens and Weaver, 2009).

### Cortical Plasticity of Spared Sensory Cortices

In animal models, vision loss leads to plasticity within A1 and S1. Mice deprived of vision since birth have enlarged whisker representations in S1 (Rauschecker et al., 1992). Visual deprivation from birth also results in decreased amplitude of mEPSCs in L2/3 of A1 and S1 in rodents (Goel et al., 2006), which as discussed later, may reflect a shift in processing of information from intracortical towards feedforward sources (Petrus et al., 2015). In an animal model, where visual deprivation can be done before the development of retinogeniculate connections, anatomical changes in cortical and subcortical inputs to S1 have been observed (Dooley and Krubitzer, 2019). Plasticity is not restricted to early-onset vision loss. At least in rodents, the adaptation of neural circuits in A1 and S1 has been observed even with a few days of dark exposure or bilateral lid suture (Goel et al., 2006; Jitsuki et al., 2011; He et al., 2012; Petrus et al., 2014, 2015; Meng et al., 2015, 2017; Solarana et al., 2019). Even in adult mice, a short duration of visual deprivation has been shown to trigger functional enhancement of feedforward inputs and refinement of functional circuits within A1 (Petrus et al., 2014, 2015; Meng et al., 2015, 2017). Specifically, when adult mice are subjected to 7 days of dark exposure, potentiation of synapses serving the feedforward pathway, thalamocortical inputs to L4, and subsequent L4 to L2/3 inputs, is observed in A1 (Petrus et al., 2014, 2015; Figure 2B). Potentiation of the feedforward connections is accompanied by a weakening of intracortical synapses onto L2/3 neurons of A1 (Petrus et al., 2015; Figure 2B), which manifests as a decrease in the average amplitude of mEPSCs (Goel et al., 2006; Petrus et al., 2015). Similarly, visual deprivation leads to a reduction in the average amplitude of mEPSCs in L2/3 of barrel cortex (Goel et al., 2006; He et al., 2012) but not in the frontal cortex (Goel et al., 2006), which suggests that this type of adaptation is common across the spared primary sensory cortices. The shift in synaptic strength to favor feedforward synapses in A1 with visual deprivation correlated with heightened sensitivity to sound, observed as a decrease in the threshold of A1 L4 neurons to sound (Petrus et al., 2014). In addition, a few days of visual deprivation-induced sharpening of tuning of A1 L4 neurons to sound frequency (Petrus et al., 2014), which is likely a reflection of increased inhibition from PV-interneurons to L4 principal neurons (Petrus et al., 2015). Furthermore, the short duration of visual deprivation leads to refinement of the spatial extent of connectivity within L4 and L2/3 of A1 (Meng et al., 2015, 2017), as well as sparsification of population-level coding of sound in L2/3 of A1 (Solarana et al., 2019). These adaptations involving circuit refinement are likely to maximize the coding capacity of A1 as demonstrated by computational modeling (Meng et al., 2015). Collectively, the compensatory plasticity observed in A1 with visual deprivation is consistent with the notion that A1 would be better at processing sound, which could underlie enhanced auditory discrimination abilities often observed in blind individuals (Lessard et al., 1998; Röder et al., 1999; Gougoux et al., 2004; Voss et al., 2004).

Improvement in auditory or tactile discrimination abilities reported in blind human subjects is, however, not universal and may depend on perceptual learning (Grant et al., 2000; Wong et al., 2011). This may stem from the fact that compensatory changes observed in the spared sensory cortices are dependent on their own sensory inputs (He et al., 2012; Petrus et al., 2014). Removing whiskers or deafening mice that are undergoing visual deprivation prevents synaptic plasticity changes observed in S1 barrel cortex (He et al., 2012) and A1 (Petrus et al., 2014), respectively. These findings suggest that the potentiation of feedforward inputs to the spared primary sensory cortices is likely driven by an experience-dependent synaptic plasticity mechanism, such as LTP. Consistent with this idea, deafening normal sighted mice recover LTP of thalamocortical inputs to L4 in V1 of adult mice (Rodriguez et al., 2019). Potentiation of feedforward connections is then expected to induce metaplasticity to compensate for the increased overall input activity, which would slide the synaptic modification threshold up to promote LTD (Figure 3B). This shift in the synaptic modification threshold would preferentially weaken intracortical synapses via LTD to provide homeostasis in neural activity.

One interesting aspect of compensatory synaptic plasticity observed in the spared primary sensory cortices is that it requires a less drastic loss in vision than is required for cross-modal recruitment. As discussed earlier, V1 plasticity induced by vision loss requires a complete loss of retinal inputs and is not observed with bilateral lid-suture (He et al., 2012). However, lid-suture is sufficient to induce compensatory synaptic plasticity in the spared cortex (He et al., 2012). This suggests that total loss of retinal input is required for cross-modal recruitment of V1, while a milder degradation of vision that would hinder using vision to guide behavior may trigger compensatory plasticity in the spared cortical areas. This also indicates that cross-modal recruitment and compensatory plasticity are likely induced independently. Another difference between the two plasticity mechanisms is the duration of visual deprivation required: V1 plasticity can be triggered by a shorter duration (i.e., 2 days is sufficient) of visual deprivation (Goel and Lee, 2007; Gao et al., 2010; He et al., 2012; Chokshi et al., 2019) than that required to observe plasticity in A1 and S1 (Goel et al., 2006; He et al., 2012).

While compensatory plasticity observed in A1 and S1 following vision loss is not critically tied to the plasticity in V1, it nonetheless needs to be triggered by the loss of vision. Therefore, there must be functional circuits that carry information or convey the state of visual experience to A1 and S1 to gate compensatory plasticity. There are several possible functional circuits that can provide information on vision to A1 and S1. One is via direct or indirect (*via* higher-order sensory cortices or through higher-order thalamic nucleus) functional projections between V1 and A1/S1. This may involve gating inhibition in the target A1/S1 circuit to enable plasticity. A second possibility is through neuromodulatory systems since the loss of vision would likely change the global arousal or attentional state of an individual to the spared sensory stimuli. A third possibility is via a bottom-up “spot-light” attentional control within each spared modality.

### Intracortical Circuits That Can Mediate Compensatory Plasticity

As mentioned in a previous section (section 2.2), there are direct cortico-cortical connections between the primary sensory cortices, and there is evidence that this functional pathway can gate plasticity. In gerbils, a direct connection from V1 gates the critical period plasticity in A1, where early eye-opening leads to termination of the critical period for A1 plasticity while delayed eye opening extends it (Mowery et al., 2016). While this study did not determine how the direct functional input from V1 gates plasticity of the feedforward circuit in A1, the observation that visual deprivation can extend the critical period is consistent with other studies demonstrating recovery of thalamocortical plasticity in the adult primary sensory cortices with cross-modal sensory deprivation (Petrus et al., 2014; Rodríguez et al., 2018).

In addition to the direct projections, feedback from higher-order sensory cortices or multisensory cortical areas also can provide information on visual experience to the spared primary sensory cortices either through direct cortico-cortical connections or indirect connections via the higher-order thalamus. As explained previously, both cortico-cortical and trans-thalamic connections arrive through L1 and influence the inhibitory circuits present in L2/3 (Ibrahim et al., 2016; Roth et al., 2016; Zhou et al., 2017). It is well documented that inhibitory circuits are well poised to gate cortical plasticity (Jiang et al., 2005). In the S1 barrel cortex, input from POm, a higher-order somatosensory thalamus, is critical for gating potentiation of whisker inputs to L2/3 (Gambino et al., 2014). In particular, POm activation generates NMDAR-mediated dendritic plateau potentials in the principal neurons in L2/3, which are necessary for the observed LTP (Gambino et al., 2014). A follow-up study demonstrated that POm gating of L4 to L2/3 LTP in the S1 barrel cortex is due to disinhibition of L2/3 principal neurons *via* activation of VIP- and PV-interneurons and a concomitant decrease in SOM-interneuron activity (Williams and Holtmaat, 2019). These studies suggest that POm activity stimulates VIP-interneurons, which in turn inhibit SOM-interneurons. SOM-interneurons are known to target inhibition to dendrites (Tremblay et al., 2016). Hence, reduced SOM-interneuron activity would cause disinhibition of dendrites of L2/3 principal neurons, which could support the activation of NMDAR-mediated dendritic plateau potentials to induce LTP of the feedforward synapses from L4. As mentioned before (see “Subcortical Sources of Inputs to V1 L2/3 That Can Mediate Cross-modal Recruitment” section), trans-thalamic connections through higher-order thalamic nuclei can transmit multisensory information to primary sensory cortices. In particular, we discussed evidence on how LP conveys visual information to A1 to modulate auditory responses (Chou et al., 2020). Whether such a functional circuit involving higher-order thalamic nuclei could mediate compensatory plasticity upon loss of vision will need to be examined.

### Thalamic Circuits That May Gate Compensatory Plasticity

Considering that compensatory plasticity of feedforward circuits in A1 and S1 depends on their respective sensory inputs (He et al., 2012; Petrus et al., 2014), there is also a possibility that gating of this plasticity could occur at the level of the thalamus. The TRN is a thin band of inhibitory neurons that surrounds and projects to the primary sensory thalamic nuclei, controlling information flow to the primary sensory cortex (Halassa and Acsády, 2016; Crabtree, 2018). Although TRN is divided roughly according to modality, about 25% of TRN cells receive multimodal input from multiple relay centers in the thalamus (Lam and Sherman, 2011; Kimura, 2014). These multisensory TRN neurons could play a role in regulating feedforward excitatory thalamocortical input to A1 and S1 based on visual experience. There is potential for multisensory TRN neurons to fire less upon vision loss, which leads to disinhibition of auditory (MGBv) and somatosensory (VPM) thalamic nuclei. This would increase feedforward activity propagation to A1 and S1, which could be the basis for driving potentiation of thalamocortical synapses in L4 as observed following visual deprivation (Petrus et al., 2014).

Another potential mode by which TRN can gate activity through the spared primary thalamic nucleus is *via* feedback projections from the respective spared primary sensory cortex. Corticothalamic L6 neurons provide feedback control of their respective primary sensory thalamic nuclei via direct excitation and disynaptic inhibition through the TRN. It was demonstrated in the somatosensory system of rodents that the feedback control is activity-dependent, such that low-frequency activation of L6 neurons in the barrel cortex predominantly inhibits VPM while higher frequency stimulation leads to activation (Crandall et al., 2015). This effect was due to the difference in short-term dynamics of excitation vs. inhibition; excitatory synaptic transmission displays facilitation while inhibitory synaptic transmission undergoes depression with a train of stimulation (Crandall et al., 2015). As mentioned previously (“Cortical Plasticity of Spared Sensory Cortices” section), one of the main adaptations of the spared cortical circuit is the potentiation of feedforward synapses (Petrus et al., 2014, 2015; Rodríguez et al., 2018; Figure 2B). Therefore, there is potential for L6 to convey the heightened cortical activity, which can result in further amplification of the spared sensory input at the level of the primary sensory thalamus.

It is important to note that increasing activity of thalamocortical inputs alone cannot support potentiation. It is known that stimulation of thalamocortical inputs to L4 in cortical slices is unable to induce LTP beyond the early critical period (Crair and Malenka, 1995; Jiang et al., 2007; Barkat et al., 2011; Rodríguez et al., 2018). In contrast, electrically stimulating dLGN *in vivo* can produce LTP in adult V1 (Heynen and Bear, 2001), which suggests that there may be additional factors present in an intact *in vivo* circuitry that may allow LTP at thalamocortical synapses in the adult cortex.

### Neuromodulatory Control of Compensatory Plasticity

As discussed in the context of cross-modal recruitment, neuromodulators play a critical role in enabling plasticity in the primary sensory cortices, even in adults. There are reports that the levels of serotonin and norepinephrine are relatively higher in spared cortices than deprived cortex following visual deprivation (Qu et al., 2000; Jitsuki et al., 2011). As will be discussed in more detail below, VIP-interneuron mediated disinhibitory circuit seems a key circuit component that can be recruited for neuromodulatory control of compensatory plasticity, in addition to cortical and subcortical control, following the loss of vision.

Loss of vision could increase the behavioral relevance or salience of the remaining sensory inputs (De Heering et al., 2016). This suggests that auditory or somatosensory inputs may be more likely to be paired with acetylcholine or norepinephrine release based on the heightened attention and/or arousal to these sensory inputs in the absence of vision. Acetylcholine is particularly interesting as a candidate for mediating compensatory plasticity because it has been observed to facilitate potentiation of feedforward thalamocortical inputs especially in adult primary sensory cortices (Dringenberg et al., 2007; Chun et al., 2013). Furthermore, there is evidence that acetylcholine can differentially alter the strength of thalamocortical and intracortical synapses, such that only the former is potentiated by nicotinic acetylcholine receptor (nAChR) activation while both inputs depress when muscarinic acetylcholine receptors (mAChRs) are activated (Gil et al., 1997). Such dual action of acetylcholine is proposed to refine A1 tuning by enhancing responses from the feedforward thalamocortical receptive field while suppressing lateral intracortical inputs (Metherate, 2011). Therefore, acetylcholine could in principle coordinate potentiation of thalamocortical synapses and depression of intracortical synapses, as well as refinement of tuning properties, observed in A1 following visual deprivation (Petrus et al., 2014, 2015). Acetylcholine is widely viewed as setting the arousal level because the activity of acetylcholine neurons in nucleus basalis is associated with a desynchronized electroencephalogram (EEG) pattern, generally accepted to indicate heightened attention (Metherate et al., 1992). It is interesting to note that during strongly desynchronized EEG activity, acetylcholine preferentially activates L1 interneurons and VIP cells by acting on nAChRs expressed on these neurons (Alitto and Dan, 2012). This would disinhibit principal neurons, potentially allowing for plasticity. On the other hand, lower levels of cortical desynchronization preferentially activate PV interneurons *via* mAChRs (Alitto and Dan, 2012), which would enhance inhibition in the circuit. This observation suggests that the degree of attention or behavioral alertness may factor into how cortical circuits undergo plasticity.

The norepinephrine system has been shown to impact network activity and plasticity in sensory cortices (Salgado et al., 2016). For example, iontophoretic application of norepinephrine to A1 of awake rodents causes A1 neurons to exhibit a greater degree of frequency selectivity (Manunta and Edeline, 1997, 1999). This is reminiscent of the sharpened frequency selectivity of A1 L4 neurons following visual deprivation (Petrus et al., 2014). It has been shown that norepinephrine acting through beta-adrenergic receptors facilitates the induction of LTP and suppresses LTD (Seol et al., 2007; Huang et al., 2012). Beta-adrenergic receptors have a lower affinity to norepinephrine compared to alpha-adrenergic receptors (Salgado et al., 2016). Therefore, higher noradrenergic tone in the spared cortical area accompanying visual deprivation (Qu et al., 2000) could activate these receptors and encourage potentiation of feedforward circuits in A1.

Among the neuromodulators discussed here, serotonin has the most concrete evidence to support a role in compensatory plasticity. As mentioned in a previous section, serotonin is critical for recovering adult cortical plasticity (Maya Vetencourt et al., 2008) and cross-modal recruitment (Lombaert et al., 2018). Of relevance to compensatory plasticity, which involves recovering thalamocortical LTP in adults (Rodríguez et al., 2018), certain serotonin receptor antagonists can block thalamocortical LTP in anesthetized rats (Lee et al., 2018). Furthermore, there is direct evidence that serotonin is specifically involved in the cross-modal compensatory plasticity of the feedforward circuit. In rats that were visually deprived via bilateral lid suture, serotonin levels were elevated in the barrel cortex, but not in V1 (Jitsuki et al., 2011). Elevated serotonin levels triggered the insertion of AMPA receptors into the synapse between L4 and L2/3 cells, enhancing feedforward processing of whisker information after visual deprivation (Jitsuki et al., 2011). How serotonin levels increase specifically in deprived (Lombaert et al., 2018) vs. spared sensory cortices (Qu et al., 2000; Jitsuki et al., 2011) is unclear, but could be due to differences in the visual deprivation paradigm. Lombaert and colleagues used monocular enucleation, while Jitsuki and colleagues performed bilateral lid-suture. As mentioned previously, lid-suture is ineffective at driving changes in V1 but induces plasticity in S1 (He et al., 2012). In any case, these studies highlight the importance of the serotonergic system in coordinating cross-modal plasticity in adults.

### Functional Circuits for Bottom-Up “Spotlight” Attentional Control of Compensatory Plasticity

A great deal of interest has been devoted recently to the concept of an attentional spotlight, also referred to as selective attention or feature-based attention. The attentional spotlight, which in higher mammals has been described as a neocortical attribute, also heavily relies on subcortical mechanisms for directing attention and cognitive resources towards one salient stimulus or modality, while de-emphasizing others (Saalmann and Kastner, 2011; Halassa and Kastner, 2017; Krauzlis et al., 2018). Global neuromodulatory systems are likely enabling factors for compensatory plasticity, while continued sensory input and spotlight attentional mechanisms may play an instructive role to shape the plasticity in the spared sensory cortices. Spotlight attention is thought to act at a subcortical level to gate the information ascending to the cortex, hence controlling the flow of activity necessary for inducing activity-dependent plasticity. Therefore, turning the attentional spotlight towards auditory and somatosensory inputs in response to visual deprivation would heighten or alter the pattern of activity reaching A1 and S1 in such a way as to drive plasticity. As mentioned before, instructive mechanisms, such as increased sensory gating, cannot alone result in plasticity at synapses that have a defined critical period for plasticity, such as the thalamocortical synapses (Crair and Malenka, 1995; Jiang et al., 2007; Barkat et al., 2011; Rodríguez et al., 2018). Therefore, especially in adults, we believe attentional spotlight mechanisms would need to work together with enabling factors, such as neuromodulators, to reopen plasticity. Indeed, prior work examining adult plasticity has noted the importance of attention and behavioral relevance in enabling plasticity (e.g., Polley et al., 2006). Here, we will highlight two potential substrates for attentional spotlight regulation of feedforward circuit plasticity involved in compensatory plasticity: superior colliculus (SC) and mediodorsal nucleus (MD). TRN is another candidate to gate sensory input, as was discussed earlier (“Thalamic Circuits That May Gate Compensatory Plasticity” section).

Superior colliculus (SC) is an evolutionarily old part of the brain which processes sensory input and computes a saliency map of the environment (Krauzlis et al., 2013). As discussed above (see “Subcortical Sources of Inputs to V1 L2/3 That Can Mediate Cross-modal Recruitment” section), SC has long been appreciated to participate in visual processing but also harbors multimodal cells in the deeper layers which integrate tactile, visual, and auditory stimuli (Krauzlis et al., 2013; Cang et al., 2018). These multimodal cells in deep layers of SC account for the majority of output neurons (Cang et al., 2018), sending collaterals to many structures, including higher-order thalamic nuclei as well as TRN (Krauzlis et al., 2013). SC input to POm, a higher-order somatosensory thalamus, has been shown to allow attentional enhancement of somatosensory stimuli in the cortex, as observed by enhanced S1 responses to weaker whisker stimulation upon activation of SC neurons (Gharaei et al., 2020). This effect may be mediated by the aforementioned disinhibition of L2/3 principal neurons upon POm activation (Williams and Holtmaat, 2019) (see “Intracortical Circuits That Can Mediate Compensatory Plasticity” section). SC also has been shown to sharpen A1 processing *via* its connections to LP (Chou et al., 2020; see “Subcortical Sources of Inputs to V1 L2/3 That Can Mediate Cross-modal Recruitment” section). Therefore, SC is in a prime position to provide multisensory information to a key circuit motif involving higher-order thalamic nuclei that can mediate localized enhancement of response properties in primary sensory cortices.

The mediodorsal nucleus (MD) is a higher-order thalamic nucleus considered to be important in attention and learning (Mitchell and Chakraborty, 2013; Mitchell, 2015), in part due to its extensive and reciprocal connections with the prefrontal cortex (Zikopoulos and Barbas, 2007; Mitchell and Chakraborty, 2013; Mitchell, 2015). In addition, MD projects to all parts of the TRN, which differs from primary thalamic nuclei which have projections mainly limited to a subregion of TRN (Zikopoulos and Barbas, 2007; Mitchell, 2015). These features suggest that MD may provide a functional connection between prefrontal cortical networks involved in the attentional selection and TRN to gate sensory input (Zikopoulos and Barbas, 2007; Mitchell, 2015). The prefrontal cortex has been shown to modulate performance on a multimodal attentional task via its effect on TRN activity (Wimmer et al., 2015). The close connection between MD and TRN thus offers a potential substrate for attentional regulation of input from the thalamus to primary sensory cortices.

## Conclusions

Primary sensory cortices are highly interconnected to multisensory cortical and subcortical structures, which under normal circumstances provide contextual and saliency information needed for proper sensory processing. We suggest that these cortical and subcortical functional connections play a critical role in mediating cross-modal plasticity when a sensory modality is lost, such that an organism can effectively navigate its environment based on the remaining senses. As summarized in this review, these functional connections will allow cross-modal recruitment of the deprived sensory cortex for processing the spared sensory information, as well as enabling and instructing plasticity needed for refining sensory processing of the spared sensory cortices. Visual-deprivation studies highlight the involvement of Hebbian and homeostatic metaplasticity in sculpting the cortical circuits for cross-modal plasticity, which involves not only the plasticity of excitatory synapses, but also that of inhibitory synapses. Cross-modal plasticity across sensory cortices is likely coordinated globally via direct connection across sensory cortices, indirect connectivity through cortico-thalamo-cortical loops or indirect cortical connections through multisensory cortical areas. It is likely that global neuromodulatory systems are engaged to enable plasticity across the sensory cortices. In parallel, multisensory functional inputs that target cortical inhibitory circuits could also gate plasticity within each cortical area. Instructive signals for plasticity likely arise through activity from cortical and subcortical multisensory inputs to V1 and feedforward inputs to the spared cortices. The latter may involve subcortical structures that provide “spotlight” attention to sculpt the spared cortices to better process the most relevant information. While future studies are needed to clarify the role of these diverse functional circuits in cross-modal plasticity, this extensive network of functional connectivity highlights the rich array of contextual information that can influence sensory processing even at the level of primary sensory cortices.

## Author Contributions

GE, SP, and H-KL wrote the manuscript. AL and YJ compiled information used for the text and generated the figures with the help of GE, SP, and H-KL. All authors contributed to the article and approved the submitted version.

## Conflict of Interest

The authors declare that the research was conducted in the absence of any commercial or financial relationships that could be construed as a potential conflict of interest.

## Abbreviations

5HT: 5-hydroxytryptamine
A1: primary auditory cortex
ACg: anterior cingulate cortex
AChR: acetylcholine receptor
AL: anterolateral area
aLP: anterior-ventral lateral posterior nucleus
AM: anteromedial area
AMPA: α-amino-3-hydroxy-5-methyl-4-isoxazolepropionic acid
BCM: Bienenstock-Cooper-Monroe
cAMP: cyclic adenosine 3, 5-monophosphate
dLGN: dorsal lateral geniculate nucleus
EEG: electroencephalogram
EPSC: excitatory postsynaptic current
fMRI: functional magnetic resonance imaging
GAD65: glutamic acid decarboxylase 65
GluN2B: glutamate receptor N-methyl-D-aspartate receptor subunit 2B
HVA: higher order visual areas
IC: intracortical
IPSC: inhibitory postsynaptic current
L1: layer 1
L2/3: layer 2/3
L4: layer 4
L5: layer 5
L6: layer 6
LD: lateral dorsal nucleus
LI: laterointermediate area
LM: lateromedial area
LP: lateral posterior nucleus
LSD: lysergic acid diethylamide
LTD: long-term depression
LTP: long-term potentiation
mAChR: muscarinic acetylcholine receptor
MD: mediodorsal nucleus
mEPSC: miniature excitatory postsynaptic current
mIPSC: miniature inhibitory postsynaptic current
MGBv: ventral division of medial geniculate body
mLP: medial lateral posterior nucleus
nAChR: nicotinic acetylcholine receptor
NMDAR: N-methyl-D-aspartate receptor
NTSR1: neurotensin receptor 1
ODP: ocular dominance plasticity
PFC: prefrontal cortex
PLC: phospholipase C
pLP: posterior-dorsal lateral posterior nucleus
PM: posteromedial area
PO: posterior thalamic nucleus
POm: posterior medial thalamic nucleus
POR: postrhinal area
PV: parvalbumin
RL: rostolateral area
RSP: retrosplenial cortex
S1: primary somatosensory cortex
SC: superior colliculus
SOM: somatostatin
STDP: spike timing dependent plasticity
TMS: transcranial magnetic stimulation
TRN: thalamic reticular nucleus
TTX: tetrodotoxin
V1: primary visual cortex
V2: secondary visual cortex
V2L: lateral secondary visual cortex
VEPs: visually evoked potentials
VIP: vasoactive intestinal peptide
VPM: ventral posteromedial nucleus.
